# Spatial and Temporal Variations of PM_2.5_ and Its Relation to Meteorological Factors in the Urban Area of Nanjing, China

**DOI:** 10.3390/ijerph13090921

**Published:** 2016-09-16

**Authors:** Tao Chen, Jun He, Xiaowei Lu, Jiangfeng She, Zhongqing Guan

**Affiliations:** 1 Jiangsu Provincial Key Laboratory of Geographic Information Science and Technology, School of Geographic and Oceanographic Sciences, Nanjing University, Nanjing 210023, China; chrisplum@smail.nju.edu.cn (T.C.); zhongqingguan@gmail.com (Z.G.); 2 Nanjing Information Center, Nanjing 210019, China; hejun@njnet.gov.cn; 3 School of the Environment, Nanjing University, Nanjing 210023, China; glxiaowei123@163.com

**Keywords:** PM_2.5_, spatial and temporal variations, meteorological factors, CEEMDAN, Nanjing

## Abstract

The serious air pollution problem has aroused widespread public concerns in China. Nanjing city, as one of the famous cities of China, is faced with the same situation. This research aims to investigate spatial and temporal distribution characteristics of fine particulate matter (PM_2.5_) and the influence of weather factors on PM_2.5_ in Nanjing using Spearman-Rank analysis and the Complete Ensemble Empirical Mode Decomposition with Adaptive Noise (CEEMDAN) method. Hourly PM_2.5_ observation data and daily meteorological data were collected from 1 April 2013 to 31 December 2015. The spatial distribution result shows that the Maigaoqiao site suffered the most serious pollution. Daily PM_2.5_ concentrations in Nanjing varied from 7.3 μg/m^3^ to 336.4 μg/m^3^. The highest concentration was found in winter and the lowest in summer. The diurnal variation of PM_2.5_ increased greatly from 6 to 10 a.m. and after 6 p.m., while the concentration exhibited few variations in summer. In addition, the concentration was slightly higher on weekends compared to weekdays. PM_2.5_ was found to exhibit a reversed relation with wind speed, relative humidity, and precipitation. Although temperature had a positive association with PM_2.5_ in most months, a negative correlation was observed during the whole period. Additionally, a high concentration was mainly brought with the wind with a southwest direction and several relevant factors are discussed to explain the difference of the impacts of diverse wind directions.

## 1. Introduction

In recent years, the air pollution problem brought by rapid economic and unprecedented urbanization construction in China has aroused widespread public concerns, especially the fine particulate matter (PM_2.5_) pollution, owing to the potential threats to health [1,2,3]. High PM_2.5_ concentration played an important role in the emergence of haze [4], which may account for the occurrence of extreme haze events in China in recent years [5,6]. Since aerodynamic diameter is less than 2.5 μm, PM_2.5_ can stay in the air for a long time and attach to harmful substances. Hence, long time exposure to PM_2.5_ concentration can lead to a significant impact on human health or even mortality [7,8,9,10,11].

Recently, a large number of studies paid attention to air suspended particulate matter (PM), especially PM_2.5_, including emission sources, physical characteristics, and chemical decomposition [12,13,14,15,16]. A better and clearer understanding of spatial and temporal variations of PM_2.5_ can contribute to the adoption of effective measures to reduce air pollution. Most research on the spatial and temporal distribution of PM_2.5_ were obtained from a remote sensing map in China [17,18]. However, real-time monitoring data is essential to better obtain the detailed variations (seasonal, monthly, and diurnal) on the city scale. For example, Zhang et al. investigated the spatial-temporal PM_2.5_ distribution characters of 190 cities in China [19]; Wang et al. analyzed the spatial and temporal variations of air pollutants at the 31 provincial capital cities of China [20]. Several studies have confirmed that meteorological variables play an important role in the formation of PM_2.5_ as well as other factors like population and emissions etc. For instance, Zhang et al. explored spatiotemporal patterns of PM_2.5_ and the determinants in 190 Chinese cities [21]. Yan et al. and Huang et al. also observed spatial and temporal variations of air pollutants including PM_2.5_ and the relationship with meteorological factors [22,23].

The Yangtze River Delta (YRD), one of the most developed and fastest urbanized areas in China, has been facing a serious air pollution problem for the past ten years [24,25]. Air quality condition is also not optimistic in Nanjing city that is located in the western region of YRD. Deng et al. found that the annual average visibility in Nanjing was just 8.8 km and a daily average value of less than 10 km occurred on 57.9% of the days in 2004 [26]. According to the statistics of the meteorological department in Nanjing, the occurrence of haze days was 242 days in 2013, reaching the highest value in historical meteorological records [27]. In addition, rare rose red smog and haze phenomenon in the winter of 2015 attracted national attention for PM_2.5_ pollution [28]. In Nanjing, many studies involving PM_2.5_ mainly focused on heavy metals and chemical decomposition [29,30,31]. However, there were very limited studies that paid attention to the spatial-temporal distributions of PM_2.5_ and its relation to meteorological conditions in the city. Shen et al. reported that the 24 h average PM_2.5_ varied from 33 μg/m^3^ to 234 μg/m^3^ at a residential site in 2012 [32]. The purpose of this paper is to comprehensively explore the spatial-temporal variations of PM_2.5_ and relationships between PM_2.5_ and meteorological factors in Nanjing, using Spearman-Rank analysis and a new time-series data decomposition method: Complete Ensemble Empirical Mode Decomposition with Adaptive Noise (CEEMDAN).

## 2. Materials and Methods

### 2.1. Site Description and Data

**Figure 1:**
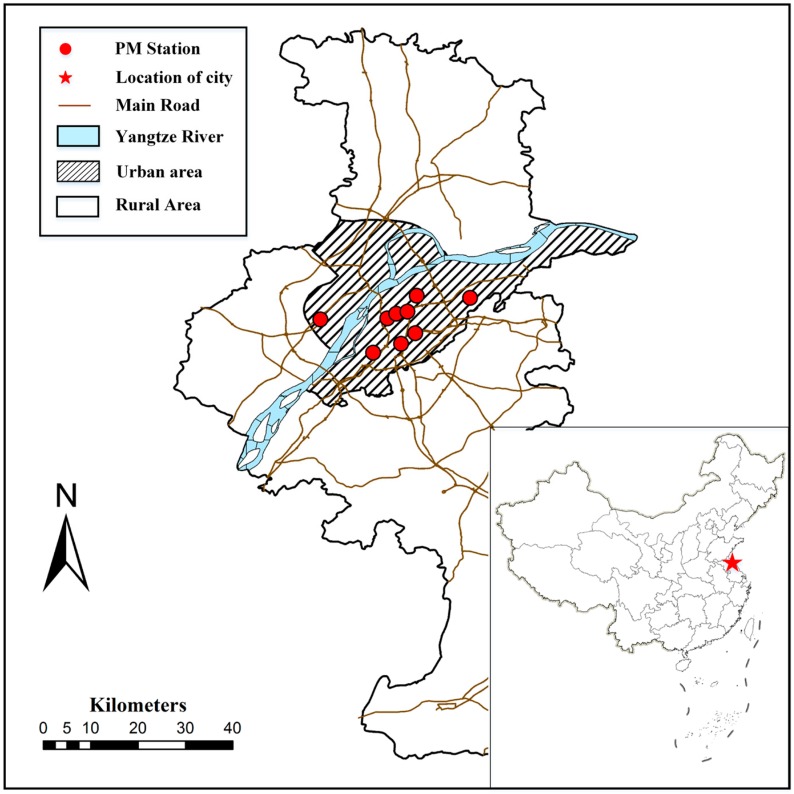
Map depicting the location of Nanjing in China (red star), air quality stations (red circles) and the distribution of main roads, urban areas, and rural areas. The corresponding PM_2.5_ monitoring sites are: Pukou, Caochangmen, Shanxilu, Xuanwuhu, Maigaoqiao, Xianlindaxuecheng, Aotizhongxin, Zhonghuamen, and Ruijinlu (from left to right: six above and three below).

Nanjing, the capital city of Jiangsu Province, is one of the ancient cities in China and has a rich historical and cultural heritage. As an intense industrialized and urbanized city with a forest coverage rate of over 35%, Nanjing is still facing a serious environmental pollution problem. There are nine monitoring sites defined by the China Environmental Monitoring Center (CEMC). All the monitoring sites are located in the urban area of Nanjing (Figure 1). Therefore, the research was carried out in the urban area. The hourly monitoring data of PM_2.5_ used in the study were collected from the CEMC [33] from 1 April 2013 to 31 December 2015. In addition, the daily meteorological data were obtained from the China Meteorological Data Network [34] during the same period, including wind speed, temperature, relative humidity, precipitation, and wind direction.

### 2.2. Methods

#### 2.2.1. Analysis of Spatial and Temporal Variations

ArcGIS [35] and cluster analysis [36] were usually carried out to explore the spatial variation of PM concentration. ArcGIS is simpler and more intuitive while the cluster analysis method requires repeatedly looking for the optimal cluster numbers. In this paper, ArcGIS was adopted to exhibit the region distribution of PM_2.5_ mass concentration in Nanjing. Mean concentration of each site of the studied period (1 April 2013 to 31 December 2015) was calculated, and ArcGIS 10.2 software (Esri, Redlands, CA, USA) was utilized to explore the distribution of PM_2.5_ in the urban area of Nanjing. Moreover, data processing was performed to obtain seasonal, monthly, and daily variations of PM_2.5_ concentrations. In addition, a valid check on hourly data was conducted to get rid of the problematic data points before data processing tasks, and all data processing tasks above were conducted with Python. 

#### 2.2.2. CEEMDAN Decomposition

Complete Ensemble Empirical Mode Decomposition (CEEMDAN) is a new powerful decomposition and analysis method for time series data by Torres et al. [37]. It is able to better decompose the input signal into a collection of intrinsic mode functions (IMFs) without prior knowledge. Each decomposition component includes specific timescale information, where the residual IMF represents the trend of the signal data. Furthermore, CEEMDAN method was improved by Ensemble Empirical Mode Decomposition (EEMD) [38] that solved the mode mixing problem of Empirical Mode Decomposition (EMD) [39] and can better decompose different IMFs in frequency scale with the best completeness and robustness. Although CEEMD is similar to Fourier analysis and wavelet decomposition, it could be used more simply and easily. This is because Fourier analysis requires sine and cosine function selection and wavelet analysis requires a wavelet basis.

According to EMD [39], each IMF should meet two conditions: (1) the difference of the number of zero-crossings related with the number of extrema must be no more than one; and (2) the average value of the envelopes computed by the local maxima and the local minima respectively is zero. The CEEMDAN method was carried out using the libeemd package [40], and the IMFs and residue of the daily PM_2.5_ concentration was calculated by performing the following steps:

- Initialize signal *x*(*t*) + *ε*_0_ *w^i^*(*t*), and decompose it by EMD to obtain their *IMF*_1_, where *x*(*t*) is the daily PM_2.5_ mass concentration series and *w*(*t*) is the appended white noise.
- Compute first residue in the first cycle: *r*_1_(*t*) = *x*(*t*) – *IMF*_1_(*t*).
- Decompose *i*-th *r*_1_(*t*) + *ε*_1_ *E_1_*(*w^i^*(*t*)), until their *IMF*_1_ of EMD and define *IMF*_2_, where *E_j_*(.) produce the *j*-th mode obtained by EMD for the given signal.
- Calculate *k*-th residue by *r_k_*(*t*) = *r_(k−1)_*(*t*) – *IMF_k_*(*t*).
- Decompose *i*-th *r_k_*(*t*) + *ε_k_ E_k_*(*w^i^*(*t*)) until their *IMF*_1_ of EMD and define (*k* + 1)-th *IMF*.
- Repeat step d for the next *k* until the residue *R*(*t*) has no more than two extrema.

Therefore, PM_2.5_ concentration signal *x*(*t*) can be represented as:

(1) $$x\left(t\right)=\sum_{k=1}^{K}IMF_{k}+R(t)$$

#### 2.2.3. Relationship between PM_2.5_ and Meteorological Factors

Daily average PM_2.5_ data and daily mean meteorological data during the whole studied period were used in this section. Firstly, hourly PM_2.5_ monitoring data was processed to obtain daily mean data. Secondly, considering the climate characteristics of Nanjing city, twelve months were divided into: spring (March to May), summer (June to August), autumn (September to November), and winter (December to February). Thirdly, Spearman-Rank correlation analysis was utilized to study the correlations between PM_2.5_ concentration and meteorological variables (wind speed, temperature, relative humidity, precipitation, and wind direction). The analysis was conducted in each of four seasons and different months respectively. In order to fully understand the effect of wind direction with PM_2.5_ concentration, Box-Whiskers plot was depicted to explore the relationship between the concentration and wind direction. Additionally, data filtering work was also necessary for precipitation with at least 1 mm and Spearman-Rank analysis and Box-Whiskers plot were carried out in Python with the Pandas package.

## 3. Results and Discussion

### 3.1. PM_2.5_ Data Overview

Table 1 shows the summary of daily mean PM_2.5_ concentrations for nine sites in Nanjing. The daily average concentrations in Nanjing varied from 7.3 μg/m^3^ to 336.4 μg/m^3^, with a broader distribution. The lowest concentration was found in the Xuanwuhu site while the highest value was observed in the Aotizhongxin site. The results show that each site’s median was much lower than mean, which means right-skewed distribution of PM_2.5_. According to the World Health Organization’s (WHO) recommended air quality guideline (AQG), 24 h average PM_2.5_ concentration should be less than 25 μg/m^3^, which is slightly lower than the grade-1 level (35 μg/m^3^) of China’s national ambient air quality standards [10,41]. Due to the relatively relaxed standard, interim targets (ITs) were simultaneously recommended for the developing countries, including IT-1 (75 μg/m^3^), IT-2 (50 μg/m^3^) and IT-3 (37.5 μg/m^3^) [42]. The percentage of daily average PM_2.5_ concentrations in Nanjing reaching the four recommended targets was 68.6%, 41.7%, 24.3% and 9.3%, respectively. The differences of the percentage reaching the standard for each site were not obvious except for the AQG. Xianlindaxuecheng site had highest percentage matching four targets, followed by Xuanwuhu site. Moreover, the percentages for three ITs at the Maigaoqiao site were the lowest, indicating the most serious PM_2.5_ pollution.

### 3.2. Regional Variation

**Figure 2:**
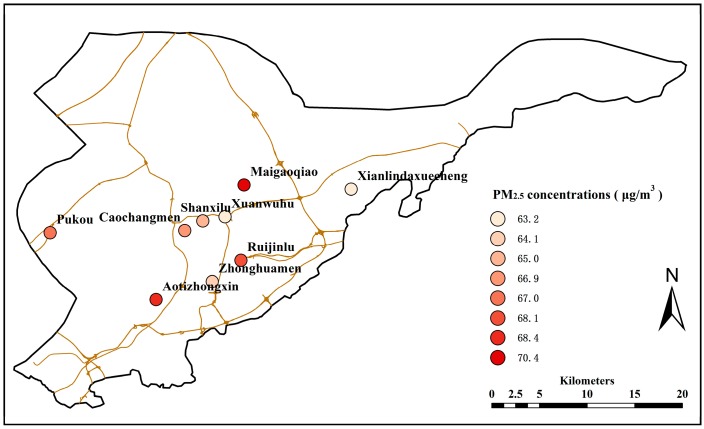
Regional distribution of the average PM_2.5_ mass concentrations in the past three years in Nanjing.

Figure 2 shows the spatial distribution of the average PM_2.5_ concentrations for each monitoring site of Nanjing in the past three years. The map shows that PM_2.5_ pollution was most serious at Maigaoqiao, followed by Aotizhongxin and Ruijinlu. The finest air quality was observed at Xuanwuhu and Xianlindaxuecheng. In order to exhaustively explain the spatial distribution of PM_2.5_, the typical characteristics of the nine monitoring sites in Nanjing were collected in Table 2. According to the information shown in Table 2, Maigaoqiao had the worst environment, and the main sources of pollution found at the Aotizhongxin site came from urban construction activities while Ruuijinlu station is located in a dense residential area; on the contrary, Xuanwuhu and Xianlindaxuecheng sites owe their superior environment to the lack of big emissions of particulate pollutants. Through the above analysis, spatial distribution was closely related to geographical location. Meanwhile, due to the systematic information of particulate matter pollution, the impact of terrain, vegetation cover, and weather conditions cannot be ignored in the process. 

### 3.3. Temporal Variation

**Figure 3:**
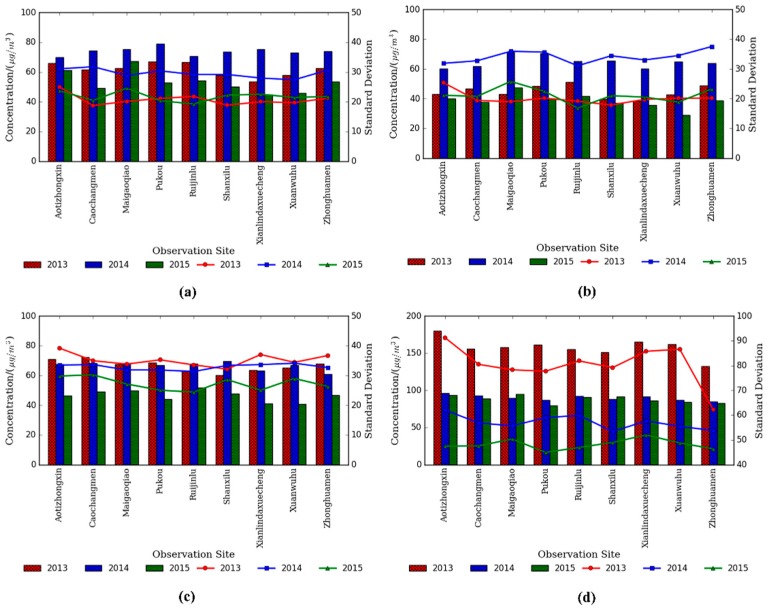
Seasonal variation of PM_2.5_ concentrations for the past three years in Nanjing. The bar represents the concentration of PM_2.5_ and the line means standard deviation; (**a**) Spring; (**b**) Summer; (**c**) Autumn and (**d**) Winter.

Seasonal variations of PM_2.5_ concentrations for all sites in Nanjing are shown in Figure 3, where April and May were only included in the spring and December was included in the winter in 2013. PM_2.5_ pollution in the winter was much more severe than other seasons, especially in 2013, with the average value of up to 158.5 μg/m^3^. Normally, it is followed by spring (63.1 μg/m^3^) and autumn (59.9 μg/m^3^). The finest air quality appeared in the summer and the concentration value in the summer of 2014 (64.8 μg/m^3^) was higher than in 2013 (44.7 μg/m^3^) and 2015 (38.8 μg/m^3^). It can be seen from the statistics of standard deviation that the pollution condition of PM_2.5_ was most turbulent in the winter of 2013, consistent with the above analysis. In addition, the concentration in 2015 was significantly lower than in 2013 or in 2014.

**Figure 4:**
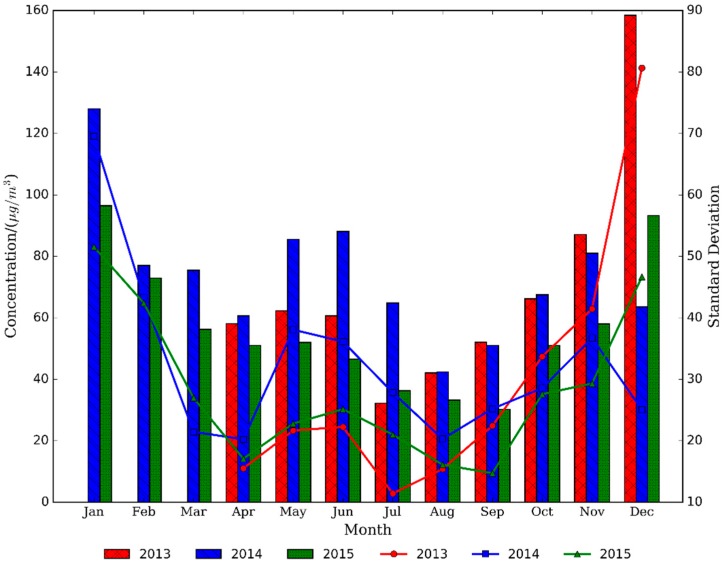
Monthly variation of PM_2.5_ concentrations in Nanjing. The bar represents PM_2.5_ concentrations and the line means standard deviation.

Figure 4 shows monthly variation of PM_2.5_ concentrations in Nanjing. For 2014, the highest PM_2.5_ concentration appeared in January. In addition, the concentration in February, March, and April continually decreased. There was a sharp rise of PM_2.5_ concentration in May, and the value in June was a little higher than in May. Subsequently, the concentration value reduced in the next two months. The lowest concentration of the whole year was observed in August, and the average value was 42.4 μg/m^3^, which is very close to the grade-1 value (32 μg/m^3^) national standard [41]. The concentration continued to increase in September, October and November. However, a rare decline occurred in December. Monthly variation in 2013 and 2015 was basically analogous to that in 2014, but several differences were observed: the lowest average value was found in July of 2013 and September of 2015; the concentration continually increased from October to December in both 2013 and 2015, different from the decrease in December of 2014; PM_2.5_ pollution was the highest for the studied period in the winter of 2013, followed by January in 2014, indicating that pollution was on going.

According to the analysis results of the Nanjing Environmental Protection Bureau [43], the main factors influencing PM_2.5_ pollution are fired coal and industrial production. Fired coal from plants and industry and dust from industrial areas and construction sites can account for the most serious pollution problems in the winter. Following the strengthening energy conservation and reduction of pollution emissions from 2014, it was unexpected to find that the concentration in December of 2015 was instead higher than that in 2014. Through checking the meteorological records, low frequency and the weak intensity of cold air in 2015 led to the above contrast.

**Figure 5:**
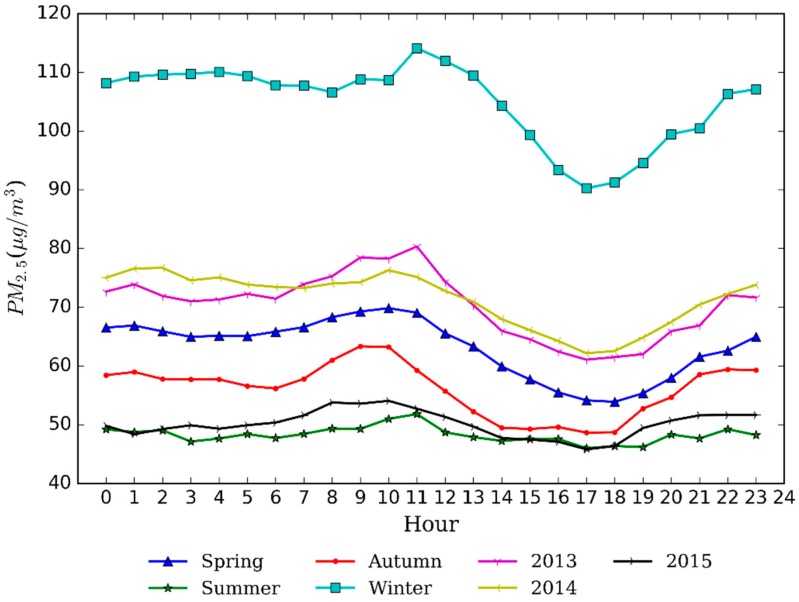
Diurnal variation of PM_2.5_ concentrations in Nanjing.

Figure 5 shows diurnal variation of PM_2.5_ for different seasons and different years in Nanjing. For seasons, the diurnal variation of PM_2.5_ concentrations in winter was higher than other seasons. It is followed by spring, autumn, and summer, which verifies the information in Figure 3. The concentration value increased significantly from 6:00 to 10:00 in the morning and after 6:00 in the evening, except in summer, where the rush hour in the morning of winter was found from 8:00 to 10:00 a.m. This means rush hour traffic emissions are of importance to PM_2.5_ concentration variation. In summer, rush hour peaked in the morning (6:00–11:00 a.m.) and PM concentration had few changes at other times of the day. In terms of years, the diurnal variation for each year showed similar trends, and PM_2.5_ concentrations of each hour in 2015 was clearly lower than in 2013 and 2014, which confirms that air quality has been improved. Due to the decline of anthropogenic emissions and favorable meteorological conditions, the value of PM_2.5_ was continually reduced from noon to afternoon/early evening. Frequent temperature inversion and the lowest height of mixing layer from the evening to the early morning are not beneficial to the vertical diffusion of pollutants [44], which contributes to the relatively high PM_2.5_ concentration in the early morning. 

**Figure 6:**
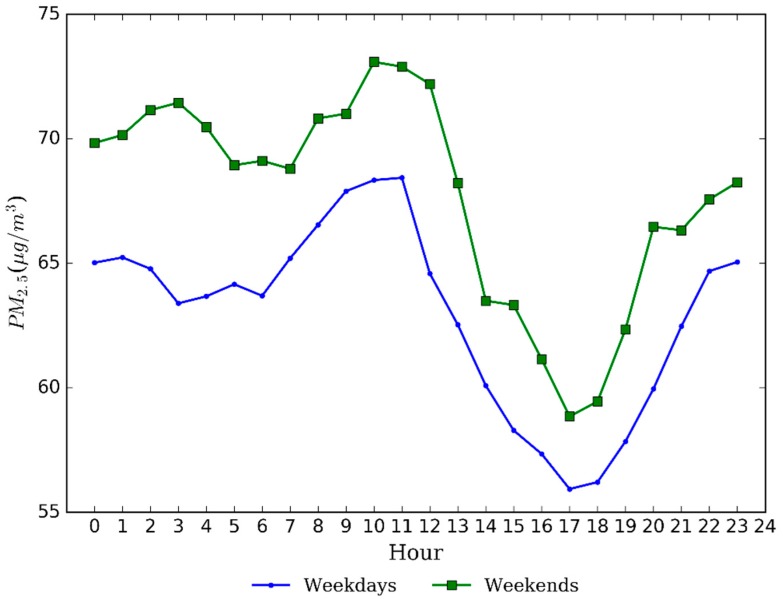
Weekday and weekend variations of PM_2.5_ concentrations in Nanjing.

Figure 6 shows average weekday and weekend variations of PM_2.5_ concentrations for the whole period in Nanjing. The mean concentration was slightly higher on weekends (67.7 μg/m^3^) than that on weekdays (63.0 μg/m^3^). This may be owing to temporary increase in the intensity of urban construction activities on weekends while those activities are restricted on weekdays. In addition, the largest differences (about 10 μg/m^3^) were only found at a few moments while most of daytime had small differences in concentration. It follows that there was no obvious variation between weekdays and weekends for PM_2.5_ concentrations in the studied period. Hu et al. also reported that PM_2.5_ concentrations in the YRD cities exhibited small variations on weekdays compared to weekends [45]. Furthermore, weekdays showed a similar trend pattern of PM_2.5_ concentration with weekends, but one hour later on weekends, indicating a change in human activities.

### 3.4. Decomposition of Time Series Data

**Figure 7:**
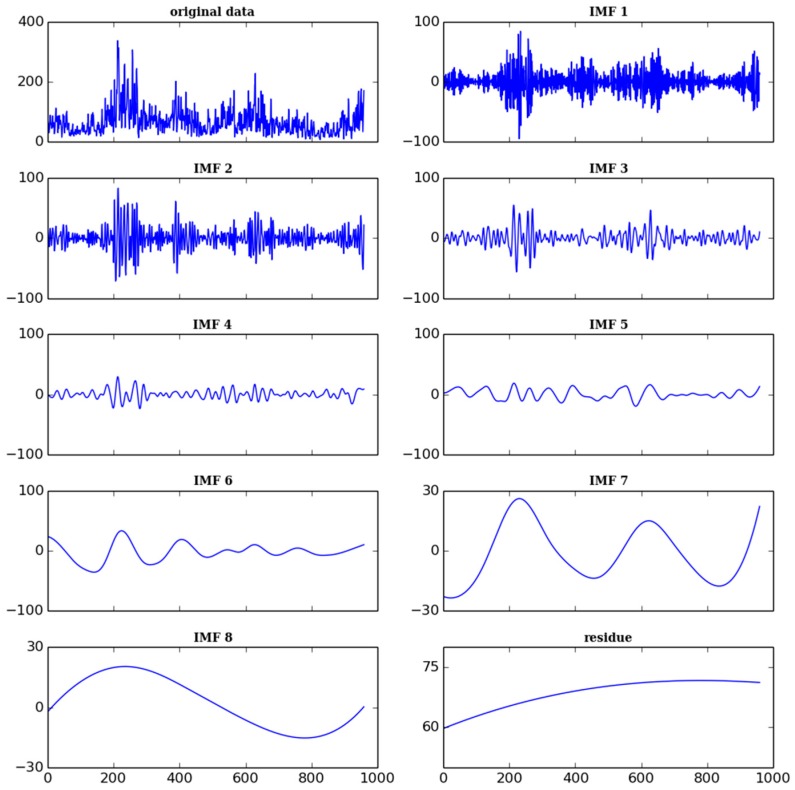
CEEMDAN decomposition of PM_2.5_ concentrations for three years.

Figure 7 shows that variations in different time frequencies and the overall trend decomposed by the given signal of PM_2.5_. Eight IMFs and one residue were obtained in the process. The results look similar but differ in amplitude and frequency. IFM1 has the highest amplitude and frequency, on the contrary, IMF8 has the lowest. When the results were stripped from the original signal, the trend of the whole period for the study was generated. As shown in the residue of Figure 7, the curve of the trend exhibits parabolic distribution. PM_2.5_ concentration in 2015 shows a slight downward trend compared with that in 2014. The result is consistent with that in the Figure 3, which indicates that measures related to environmental protection have been successful. Due to the uncomplete data, we cannot obtain the overall trend for the whole year of 2013.

**Figure 8:**
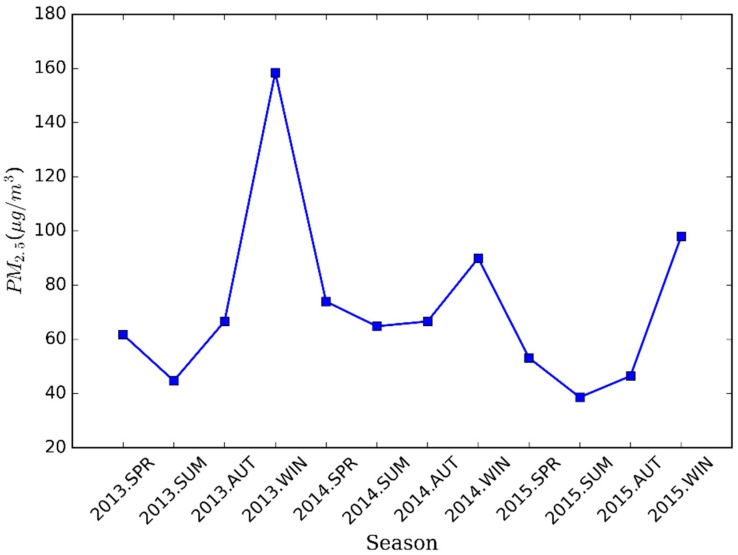
Seasonal distribution of PM_2.5_ concentrations for three years based on the CEEMDAN decomposition results.

In general, low-frequency signals contain yearly variations while high frequency signals include sudden changes. IMFs from IMF1 to IMF6 mean the period less than a year while IMF8 represents inter-annual changes. In addition, the IMF7 represents the cycle of about a year. Therefore, the original signal was reconstructed by getting together the IMFs without IMF8 and the residue to obtain seasonal changes of PM_2.5_ mass concentration in Nanjing. The mean concentration in each season was computed by averaging the reconstructed data series in the four seasons. Figure 8 shows the mean concentration in the different seasons of three years, where the data are not complete in the spring and winter of 2013. Several details can be seen: (1) the seasonal PM_2.5_ concentration varied greatly; (2) the highest concentration was always found in winter while the lowest was in summer; and (3) the concentration in winter of 2015 was a little higher than in 2014. 

Integrating the high frequency information in Figure 7, the number of days and the amplitude for sudden changes were significantly decreased in the same period. During the study period, sudden changes of PM_2.5_ concentration mainly focused on January and December, especially in December of 2013. There are a variety of factors that cause an instant increase of PM_2.5_ concentration [29,46], like fireworks, dust episodes, and gas heating. Fireworks occur mainly around the Spring Festival and lead to sudden increases in PM_2.5_ concentration in the winter. Fired coal from plants is used throughout winter while dust episodes happen occasionally throughout the whole year.

### 3.5. Correlation between PM_2.5_ and Meteorological Factors

**Figure 9:**
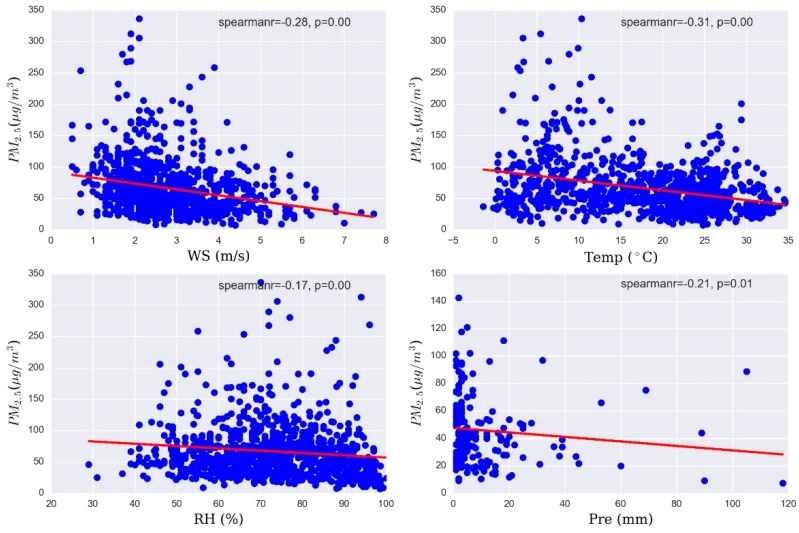
The relationship between PM_2.5_ concentrations and meteorological conditions.

Table 3 shows that the correlation coefficients of PM_2.5_ concentration related with wind speed, temperature, relative humidity, and precipitation using the Spearman-Rank analysis method. A negative relationship was weakly exhibited among PM_2.5_ and wind speed in the season months, which does not match a similar study [47]. The most possible reason is the effect of mountainous terrain on the wind in the urban area. Temperature positively correlated with PM_2.5_ in most months. This is because high temperature contributes to photochemical activity to produce more secondary particles [48]. Relative humidity had a strong negative association with PM_2.5_ in summer. Very high humidity can make suspended particles get together, then particles cannot stay in the air and fall to the ground to cause the decrease of PM_2.5_ concentrations. Precipitation showed strongly reverse correlation with PM_2.5_ in February and winter. In order to better explore the overall effects of meteorological variables, Spearman-Rank correlations between PM_2.5_ concentration and weather conditions are shown in Figure 9. Wind speed, relative humidity, and precipitation had weak negative associations with PM_2.5_ concentrations. However, a negative relationship was found between temperature and PM_2.5_ during the whole period. The above analysis results make us believe that the influence of meteorological parameters is a very complex and comprehensive process. Therefore, more detailed study is needed to analyze the impact of weather conditions on PM_2.5_ concentration in the future, such as adding hourly meteorological data and exploring multiple relationships.

### 3.6. Effect of Wind Direction on PM_2.5_ Concentration

**Figure 10:**
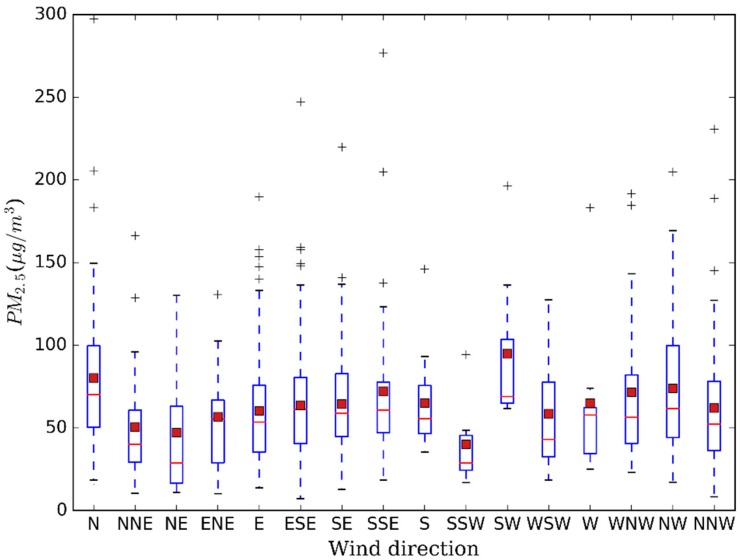
Box-Whiskers plot of PM_2.5_ mass concentration related with wind direction. Bottom and top of the blue box represent 25th and 75th percentile, whereas bottom and top of the vertical dotted line mean minimum and maximum value. The red solid lines represent median value and the firebrick square represent mean value. Outliers are depicted by the “+”.

**Figure 11:**
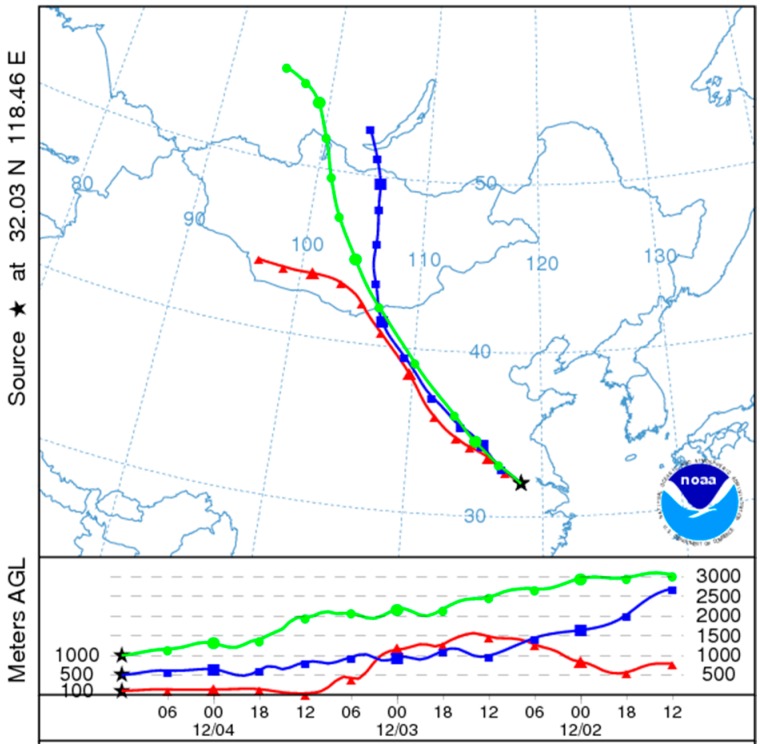
Analysis of the back trajectories of air flows for the 72 h cycle in the winter of 2014 based on the meteorological data of Global Data Assimilation System (GDAS).

As an important meteorological factor, the role of wind direction cannot be ignored [49]. PM_2.5_ concentration related with specific wind direction is presented in Figure 10. The figure shows that southwest wind led to the highest PM concentration, followed by north wind and northwest wind. Remarkably, such a result is not consistent with what people had expected, that is, owing to the industry pollution in the north region [20], the winds from north were expected to lead to high PM_2.5_ concentration, meanwhile southwest wind should not. However, the results were not as expected and there is a slight difference among north winds. There are several possible aspects to be discussed about this contrast. First, there is a big iron and steel industry zone close to the urban area in the southwest of Nanjing and the base of Ma’anshan iron and steel industry is also located in the direction adjacent to Nanjing city. Therefore, large amount of particles generated from the industry can spread to urban areas by a southwest wind, which directly aggravates the PM_2.5_ pollution. Second, particles can be transported into Nanjing from the north region, which could be confirmed by the trajectories of air flows using the HYSPLIT model in Figure 11. Nonetheless, with the transportation of pollutants, cold air is usually brought by air flows directly from the north and then it encounters the warm flows from south in the Nanjing area. In the process, favorable weather conditions can be formed and directly lead to the attenuation of PM_2.5_, which cause unremarkable PM_2.5_ pollution by winds with north directions. Meanwhile, due to the mountain terrain in the northwest, it is not beneficial to the horizontal diffusion of air pollutants and leads to higher PM_2.5_ concentration related with northwest wind. Additionally, the result might partly verify the preliminary analysis of the pollution source that PM_2.5_ pollution is mainly derived from local pollution while regional transportation was responsible for about 28.5% [43]. Certainly, the analysis of wind direction involves many factors, such as pollution sources, terrain, mixing layer, etc., and due to the limited data, basic analysis is only given for the statistic result in this section. 

## 4. Conclusions

Spatial-temporal variations of PM_2.5_ and its relation with meteorological variables in Nanjing were analyzed in the paper and several conclusions can be drawn as follows:

(a) Daily average PM_2.5_ concentration varied from 7.3 μg/m^3^ to 336.4 μg/m^3^. The ArcGIS map shows that Maigaoqiao suffered the poorest air quality, followed by Aotizhongxin and Ruijinlu. The lowest concentration was found at the Xuanwuhu site. The results show that spatial distribution of PM_2.5_ is not only related to geographical location but also other factors.
(b) Seasonal variation of PM_2.5_ was obvious and the highest concentration was found in winter while the lowest occurred in summer. The diurnal PM_2.5_ concentration increased significantly from 6:00 to 10:00 in the morning and after 6:00 in the evening and stayed relatively high in the early morning. There was no significant weekend effect in Nanjing city. The CEEMDAN decomposition results show that the frequency and intensity of sudden change decreased year by year and there was a decreasing trend from 2014.
(c) Spearman-Rank analysis was involved to explore the relationship between PM_2.5_ and meteorological factors. PM_2.5_ exhibited a negative association with wind speed, relative humidity, and precipitation. Temperature positively correlated with PM_2.5_ in most months but showed a negative correlation during the whole period, indicating a complex influence. In addition, high PM_2.5_ concentration was mainly related to southwest wind.

There are still several phenomena that need comprehensive study, such as the influence of meteorological factors including mixing layer and the effect of terrain on PM concentration related to wind. It is also worth further exploring the differences of PM_2.5_ mass concentration between urban areas and rural areas in Nanjing city. The content mentioned above will be taken into account in a future study.

## Figures and Tables

**Table 1:** The summary of daily average fine particulate matter (PM_2.5_) concentrations for nine monitoring sites.

Station	Basic Statistic (μg/m^3^)					Percentage Reaching for Standard ^1^ (%)			
	Max.	Mean	Median	Min.	Std.	IT-1	IT-2	IT-3	AQG
Aotizhongxin	373.3	68.4	58.0	2.0	46.8	67.6	41.4	24.5	9.5
Caochangmen	353.1	66.9	56.9	4.0	43.4	69.6	41.4	24.5	9.0
Maigaoqiao	327.7	70.4	61.0	8.7	43.0	64.8	35.6	21.0	6.6
Pukou	336.0	67.0	57.5	2.7	42.5	67.7	40.9	23.8	7.8
Ruijinlu	338.0	68.1	58.0	9.7	42.1	69.6	40.3	23.8	4.3
Shanxilu	332.6	65.0	55.4	6.1	42.7	70.6	44.3	27.1	11.6
Xianlindaxuecheng	319.5	63.2	53.0	3.0	45.6	72.0	46.3	31.5	15.3
Xuanwuhu	366.7	63.2	54.0	1.0	44.5	70.8	46.2	29.8	14.3
Zhonghuamen	303.0	64.1	56.0	4.8	39.3	71.5	43.5	26.4	10.4

^1^ World Health Organization (WHO) (2006) recommends the air quality guideline (AQG, 25 μg/m^3^) and three interim targets (IT-1, 75 μg/m^3^; IT-2, 50 μg/m^3^; IT-3, 37.5 μg/m^3^).

**Table 2:** The typical characteristics for each particulate matter (PM) monitoring station in Nanjing city.

Station	Main Activities and Characteristics around Station
Aotizhongxin	New city area, frequent urban construction activities
Caochangmen	Adjacent to the Yangtze River, around the universities, tourism
Maigaoqiao	Once urban-rural binding region, frequent urban construction activities, petrochemical technology, thermal power plant
Pukou	Nanjing Laoshan National Forest Park (station inside), dense construction activities, tourism
Ruijinlu	Dense residential area, tourism
Shanxilu	Residential area, shopping center
Xianlindaxuecheng	University area, shopping malls
Xuanwuhu	Xuanwuhu Lake (largest city park in the Jiangnan region), tourism
Zhonghuamen	Traffic arteries, tourism

**Table 3:** The correlation coefficients of daily PM_2.5_ related with meteorological variables.

Month/Season	WS	T	RH	Pre
January	−0.07	0.53 **	−0.01	−0.43
February	−0.22	0.35 **	−0.17	−0.76 **
March	−0.06	0.43 **	0.06	−0.39
April	−0.23	0.28 *	−0.04	0.32
May	−0.41 **	0.41 **	−0.17	−0.27
June	−0.37 **	0.34 **	−0.23 *	−0.23
July	−0.36 **	−0.02	−0.06	0.05
August	−0.35 **	0.33 **	−0.45 **	0.07
September	−0.36 **	0.35 **	0.05	−0.35
October	−0.30 **	−0.03	−0.10	−0.28
November	−0.38 **	0.35 **	−0.34 **	−0.26
December	−0.16	0.29 **	0.28 **	−0.80
Winter	−0.17	0.35 **	0.02	−0.67 **
Spring	−0.26 **	0.22 **	−0.05	−0.17
Summer	−0.26 **	−0.03	−0.60 *	−0.05
Autumn	−0.38 **	−0.26 **	−0.19 **	−0.28

Note: WS, Wind Speed; T, Temperature; RH, Relative Humidity; Pre, Precipitation; * *p* < 0.05; ** *p* < 0.01.

## References

[1] Wang S., Hao J. (2012). Air quality management in China: Issues, challenges, and options. J. Environ. Sci..

[2] Han L.J., Zhou W.Q., Li W.F., Li L. (2014). Impact of urbanization level on urban air quality: A case of fine particles (PM_2.5_) in Chinese cities. Environ. Pollut..

[3] Zhang F., Cheng H.R., Wang Z.W., Lv X.P., Zhu Z.M., Zhang G., Wang X.M. (2014). Fine particles (PM_2.5_) at a CAWNET background site in Central China: Chemical compositions, seasonal variations and regional pollution events. Atmos. Environ..

[4] Liu Y.-J., Zhang T.-T., Liu Q.-Y., Zhang R.-J., Sun Z.-Q., Zhang M.-G. (2014). Seasonal variation of physical and chemical properties in TSP, PM_10_ and PM_2.5_ at a roadside site in Beijing and their influence on atmospheric visibility. Aerosol Air Qual. Res..

[5] Huang R.-J., Zhang Y., Bozzetti C., Ho K.-F., Cao J.-J., Han Y., Daellenbach K.R., Slowik J.G., Platt S.M., Canonaco F. (2014). High secondary aerosol contribution to particulate pollution during haze events in China. Nature.

[6] Zhao X., Zhao P., Xu J., Meng W., Pu W., Dong F., He D., Shi Q. (2013). Analysis of a winter regional haze event and its formation mechanism in the North China Plain. Atmos. Chem. Phys..

[7] Bell M.L., Dominici F., Ebisu K., Zeger S.L., Samet J.M. (2007). Spatial and temporal variation in PM_2.5_ chemical composition in the United States for health effects studies. Environ. Health Perspect..

[8] Dockery D.W., Pope C.A., Xu X., Spengler J.D., Ware J.H., Fay M.E., Ferris B.G., Speizer F.E. (1993). An association between air pollution and mortality in six U.S. cities. N. Engl. J. Med..

[9] Pope C.A., Burnett R.T., Thun M.J., Calle E.E., Krewski D., Ito K., Thurston G.D. (2002). Lung cancer, cardiopulmonary mortality, and long-term exposure to fine particulate air pollution. JAMA.

[10] Burden of Disease from Household Air Pollution for 2012. http://www.who.int/phe/health_topics/outdoorair/databases/FINAL_HAP_AAP_BoD_24March2014.pdf?ua=1.

[11] Chen Z., Wang J.N., Ma G.X., Zhang Y.S. (2013). China tackles the health effects of air pollution. Lancet.

[12] Xiao Z., Bi X., Feng Y., Wang Y., Zhou J., Fu X., Weng Y. (2012). Source apportionment of ambient PM_10_ and PM_2.5_ in urban area of Ningbo city. Res. Environ. Sci..

[13] Sun Y.W., Zhou X.H., Wai K.M., Yuan Q., Xu Z., Zhou S.Z., Qi Q., Wang W.X. (2013). Simultaneous measurement of particulate and gaseous pollutants in an urban city in North China Plain during the heating period: Implication of source contribution. Atmos. Res..

[14] Wang Z., Hu M., Wu Z., Yue D., He L., Huang X., Liu X., Wiedensohler A. (2013). Long-term measurements of particle number size distributions and the relationships with air mass history and source apportionment in the summer of Beijing. Atmos. Chem. Phys..

[15] Ma J., Chen Z., Wu M., Feng J., Horii Y., Ohura T., Kannan K. (2013). Airborne PM_2.5_/PM_10_-associated chlorinated polycyclic aromatic hydrocarbons and their parent compounds in a suburban area in Shanghai, China. Environ. Sci. Technol..

[16] Yuan Q., Yang L., Dong C., Yan C., Meng C., Sui X., Wang W. (2015). Particle physical characterisation in the Yellow River Delta of Eastern China: Number size distribution and new particle formation. Air Qual. Atmos. Health.

[17] Ma Z., Hu X., Sayer A.M., Levy R., Zhang Q., Xue Y., Tong S., Bi J., Huang L., Liu Y. (2015). Satellite-based spatiotemporal trends in PM_2.5_ concentrations: China, 2004–2013. Environ. Health Perspect..

[18] Ma Z., Liu Y., Zhao Q., Liu M., Zhou Y., Bi J. (2016). Satellite-derived high resolution PM_2.5_ concentrations in Yangtze River Delta Region of China using improved linear mixed effects model. Atmos. Environ..

[19] Zhang Y.L., Cao F. (2015). Fine particulate matter (PM_2.5_) in China at a city level. Sci. Rep..

[20] Wang Y.G., Ying Q., Hu J.L., Zhang H.L. (2014). Spatial and temporal variations of six criteria air pollutants in 31 provincial capital cities in China during 2013–2014. Environ. Int..

[21] Zhang H., Wang Z., Zhang W. (2016). Exploring spatiotemporal patterns of PM_2.5_ in China based on ground-level observations for 190 cities. Environ. Pollut..

[22] Yan S., Cao H., Chen Y., Wu C., Hong T., Fan H. (2016). Spatial and temporal characteristics of air quality and air pollutants in 2013 in Beijing. Environ. Sci. Pollut. Res..

[23] Huang F., Li X., Wang C., Xu Q., Wang W., Luo Y., Tao L., Gao Q., Guo J., Chen S. (2015). PM_2.5_ spatiotemporal variations and the relationship with meteorological factors during 2013–2014 in Beijing, China. PLoS ONE.

[24] Shi C., Yang J., Qiu M., Zhang H., Zhang S., Li Z. (2010). Analysis of an extremely dense regional fog event in Eastern China using a mesoscale model. Atmos. Res..

[25] Ding A., Fu C., Yang X., Sun J., Zheng L., Xie Y., Herrmann E., Nie W., Petäjä T., Kerminen V.-M. (2013). Ozone and fine particle in the western Yangtze River Delta: An overview of 1 yr data at the SORPES station. Atmos. Chem. Phys..

[26] Deng J.J., Wang T.J., Jiang Z.Q., Xie M., Zhang R.J., Huang X.X., Zhu J.L. (2011). Characterization of visibility and its affecting factors over Nanjing, China. Atmos. Res..

[27] Nanjing Meteorology Bureau. http://www.njqxj.gov.cn/.

[28] Rose-Red Haze Show Nanjing. http://www.sh.xinhuanet.com/2015-12/23/c_134943910.htm.

[29] Kong S., Li X., Li L., Yin Y., Chen K., Yuan L., Zhang Y., Shan Y., Ji Y. (2015). Variation of polycyclic aromatic hydrocarbons in atmospheric PM_2.5_ during winter haze period around 2014 Chinese Spring Festival at Nanjing: Insights of source changes, air mass direction and firework particle injection. Sci. Total Environ..

[30] Hu X., Zhang Y., Ding Z., Wang T., Lian H., Sun Y., Wu J. (2012). Bioaccessibility and health risk of arsenic and heavy metals (Cd, Co, Cr, Cu, Ni, Pb, Zn and Mn) in TSP and PM_2.5_ in Nanjing, China. Atmos. Environ..

[31] Cui F., Chen M., Ma Y., Zheng J., Yao L., Zhou Y. (2016). Optical properties and chemical apportionment of summertime PM_2.5_ in the suburb of Nanjing. J. Atmos. Chem..

[32] Shen G.F., Yuan S.Y., Xie Y.N., Xia S.J., Li L., Yao Y.K., Qiao Y.Z., Zhang J., Zhao Q.Y., Ding A.J. (2014). Ambient levels and temporal variations of PM_2.5_ and PM_10_ at a residential site in the mega-city, Nanjing, in the western Yangtze River Delta, China. J. Environ. Sci. Health Part A.

[33] China Environmental Monitoring Center. http://113.108.142.147:20035/emcpublish/.

[34] China Meteorological Data Network. http://data.cma.gov.cn/.

[35] Feng X., Li Q., Zhu Y., Wang J., Liang H., Xu R. (2014). Formation and dominant factors of haze pollution over Beijing and its peripheral areas in winter. Atmos. Pollut. Res..

[36] Baxter L.K., Sacks J.D. (2014). Clustering cities with similar fine particulate matter exposure characteristics based on residential infiltration and in-vehicle commuting factors. Sci. Total Environ..

[37] Torres M.E., Colominas M.A., Schlotthauer G., Flandrin P. A complete ensemble empirical mode decomposition with adaptive noise. Proceedings of the 2011 IEEE International Conference on Acoustics, Speech and Signal Processing (ICASSP).

[38] Wu Z., Huang N.E. (2009). Ensemble empirical mode decomposition: A noise-assisted data analysis method. Adv. Adapt. Data Anal..

[39] Huang N.E., Shen Z., Long S.R., Wu M.C., Shih H.H., Zheng Q., Yen N.-C., Tung C.C., Liu H.H. (1998). The empirical mode decomposition and the Hilbert spectrum for nonlinear and non-stationary time series analysis. Proc. R. Soc. Lond. A.

[40] Luukko P., Helske J., Räsänen E. (2016). Introducing libeemd: A program package for performing the ensemble empirical mode decomposition. Computat. Stat..

[41] Ministry of Environment Protection of China (2012). Ambient Air Quality Standards (GB3095–2012).

[42] World Health Organization (2006). Air Quality Guidelines—Global Update 2005.

[43] China MEP Expose 9 Big Cities’ Pollution Source, Nanjing Blame Fired-Coal. http://www.njhb.gov.cn/43123/201504/t20150402_3248553.html.

[44] Pastuszka J.S., Rogula-Kozłowska W., Zajusz-Zubek E. (2010). Characterization of PM_10_ and PM_2.5_ and associated heavy metals at the crossroads and urban background site in Zabrze, Upper Silesia, Poland, during the smog episodes. Environ. Monit. Assess..

[45] Hu J., Wang Y., Ying Q., Zhang H. (2014). Spatial and temporal variability of PM_2.5_ and PM_10_ over the North China Plain and the Yangtze River Delta, China. Atmos. Environ..

[46] Yang L., Gao X., Wang X., Nie W., Wang J., Gao R., Xu P., Shou Y., Zhang Q., Wang W. (2014). Impacts of firecracker burning on aerosol chemical characteristics and human health risk levels during the Chinese New Year Celebration in Jinan, China. Sci. Total Environ..

[47] Wang J., Ogawa S. (2015). Effects of meteorological conditions on PM_2.5_ concentrations in Nagasaki, Japan. Int. J. Environ. Res. Public Health.

[48] Li J.J., Wang G.H., Wang X.M., Cao J.J., Sun T., Cheng C.L., Meng J.J., Hu T.F., Liu S.X. (2013). Abundance, composition and source of atmospheric PM_2.5_ at a remote site in the Tibetan Plateau, China. Tellus B.

[49] Charron A., Harrison R.M. (2005). Fine (PM_2.5_) and coarse (PM_2.5–10_) particulate matter on a heavily trafficked London highway: Sources and processes. Environ. Sci. Technol..

